# Function-Specific Profiles of Chronic Low Back Pain in Middle-Aged and Older Patients and Their Association with Standing Sagittal Spinopelvic Alignment: A Cross-Sectional Study

**DOI:** 10.3390/geriatrics11050144

**Published:** 2026-10-07

**Authors:** Toru Morimoto, Tatsunori Ikemoto, Young-Chang Arai, Atsuhiko Hirasawa, Norimitsu Wakao

**Affiliations:** 1 Department of Pain Medicine, Aichi Medical University,1-1 Yazako Karimata, Nagakute 480-1195, Aichi, Japan; toru.morimoto1024@gmail.com (T.M.);; 2 Department of Orthopaedic Surgery, Kochi Prefectural Hata Kenmin Hospital, Sukumo 788-0785, Kochi, Japan; 3 Department of Orthopaedics, Aichi Medical University, 1-1 Yazako Karimata, Nagakute 480-1195, Aichi, Japan

**Keywords:** chronic low back pain, gait pain, geriatric functional assessment, pelvic incidence-lumbar lordosis (PI-LL) mismatch, sagittal spinopelvic alignment

## Abstract

**Background/Objectives:** Chronic low back pain (LBP) in older adults is usually quantified as a single global score, which may not reflect the activities in which pain actually limits the patient. This study described function-specific LBP profiles in middle-aged and older patients and examined their cross-sectional association with standing sagittal spinopelvic alignment. **Methods:** This cross-sectional, retrospective observational study evaluated 102 patients with chronic LBP, categorized into Group M (under 75 years; *n* = 51) and Group O (75 years or older; *n* = 51). Visual Analog Scale (VAS) scores were assessed during rest, getting up, rolling over, and walking (gait). An exploratory index, the proportional pain contribution (VAS%), was derived by expressing each phase as a percentage of the sum of the four scores; because the four values sum to 100% by definition, VAS% was interpreted only as a descriptor of the within-patient distribution of pain and always alongside the absolute scores. Lumbar lordosis (LL), pelvic incidence (PI), pelvic tilt (PT), sacral slope and PI minus LL (PI–LL) were measured on standing radiographs. **Results:** Group M reported higher absolute pain than Group O at rest, on getting up and on rolling over (all *p* < 0.001), whereas absolute gait pain did not differ between the groups (*p* = 0.730). As a consequence of this pattern, Gait VAS% was higher in Group O (median 59.8% vs. 27.3%, *p* < 0.001). Group O had lower LL (*p* = 0.001) and a larger PI–LL (*p* = 0.005); PI and PT did not differ significantly. Gait VAS% correlated with age (τ = 0.33) and PI–LL (τ = 0.29). In exploratory multivariable models, age and PI–LL were retained in the final model for Gait VAS% and were independently associated with it (β = 0.37 and 0.30 respectively; R^2^ = 0.29). **Conclusions**: In this sample, the older patients reported less pain at rest and during postural transitions but similar pain during walking, so that walking accounted for a larger share of their total reported pain burden. This proportional pattern was associated with a larger standing PI–LL mismatch, although the association was modest. The findings are descriptive and hypothesis-generating, and suggest that asking separately about pain during walking may add information that a single global score does not capture.

## 1. Introduction

Low back pain (LBP) is the most pervasive musculoskeletal disorder in contemporary society and serves as a primary determinant of diminished quality of life (QOL) across all demographics. Within an increasingly aging global population, LBP among the elderly extends beyond physical suffering; it precipitates substantial socioeconomic burdens, including escalating healthcare expenditures and heightened caregiving requirements [1,2]. Epidemiological evidence suggests that the prevalence of LBP typically peaks between the ages of 80 and 85 before slightly declining [3]. Structural spinal degeneration—intervertebral disc degeneration, facet joint hypertrophy, osteoporotic vertebral fracture and spinal stenosis—accumulates with age, yet the association between the severity of radiographic degeneration and subjective pain intensity is weak, and age-related differences in reported pain intensity vary with the assessment method and the body position in which pain is rated [4,5].

This discrepancy complicates clinical assessment. Conventional instruments such as the visual analogue scale (VAS) or the numerical rating scale usually capture “average” pain at rest or during unspecified daily activity. Work in knee osteoarthritis indicates that weight-bearing pain corresponds more closely to physical disability than resting pain does, and a similar argument applies to LBP: pain reported during distinct functional phases—resting, getting up, rolling over and walking—may carry different clinical information [5].

For cohort definition, 75 years is an established administrative and clinical boundary in Japanese geriatric medicine, beyond which the prevalence of frailty, sarcopenia and mobility limitation rises steeply; biogerontological studies of the plasma proteome have also described distinct shifts in late adulthood [6]. We therefore compared a middle-aged group (Group M, <75 years) with an older group (Group O, ≥75 years).

Sagittal spinopelvic alignment, and in particular the mismatch between pelvic incidence and lumbar lordosis (PI–LL), is well established as a correlate of physical disability, gait disturbance and reduced health-related quality of life in older adults [7,8]. Since PI–LL mismatch is known to affect walking capacity specifically, we reasoned that it might be associated not only with the magnitude of chronic LBP but with the distribution of that pain across activities. The objective of this study was therefore to describe function-specific LBP profiles in middle-aged and older patients and to examine their observational association with standing sagittal spinopelvic parameters. We emphasize at the outset that the design permits statements about association only.

## 2. Materials & Methods

### 2.1. Study Design

This was a retrospective observational study of cross-sectional associations between clinical pain profiles and standing spinopelvic parameters. Following a preliminary hypothesis and an a priori sample size calculation, eligible patients were identified from a consecutive clinical cohort attending our outpatient clinic within the study period. Standardised clinical intake data and routine standing radiographs were extracted from electronic medical records. The study was non-interventional: all data were generated during routine care and no aspect of patient management was altered. Reporting follows the Strengthening the Reporting of Observational Studies in Epidemiology (STROBE) statement for cross-sectional studies [9].

### 2.2. Ethics and Consent

All procedures involving human participants were conducted in accordance with the 1964 Declaration of Helsinki and its later amendments or comparable ethical standards. This study protocol was approved by the Institutional Review Board of Aichi Medical University Hospital. Because the study relied exclusively on pre-existing records documented during routine care, the IRB waived the requirement for individual written informed consent. The study objective and the intended use of the data were disclosed publicly through an institutional opt-out notice on the hospital website, allowing all eligible patients to decline participation [10].

### 2.3. Eligibility Criteria

#### 2.3.1. Inclusion Criteria

Patients aged 40 years or older presenting with chronic LBP as their primary complaint lasting for more than 3 months, who visited the Department of Orthopedic Surgery at Aichi Medical University Hospital between June 2024 and December 2025, were eligible for enrollment.

#### 2.3.2. Exclusion Criteria

Patients were excluded from the study if they met any of the following criteria: suspected lumbar spinal stenosis presenting with radiating neurogenic pain from the lower back to the buttocks or lower extremities; confirmed or suspected cognitive impairment; severe neurological disorders (e.g., spinal cord injury with paralysis, advanced Parkinson’s disease, or multiple sclerosis); current oncological treatment; a history of spinal surgery within the preceding six months; or severe language comprehension or communication disorders.

### 2.4. Data Collection

Clinical data were extracted from electronic medical records, including patient demographics (age, sex, and body mass index [BMI]). Pain intensity was recorded using a standardized intake questionnaire routinely administered to all new outpatients with chronic LBP at Aichi Medical University Hospital. Patients completed the questionnaire themselves at the first consultation, rating the intensity of their low back pain—explicitly, pain localised to the lumbar region rather than lower-limb symptoms—on a 100-mm VAS under four conditions: rest, getting up from a supine position, rolling over in bed, and active walking. The attending physician entered the completed scores into the electronic record. On the same questionnaire, patients indicated which one of the four situations caused their most severe low back pain; where a patient did not complete this item, the phase with the highest recorded VAS was used, and ties were resolved in favour of the more demanding phase.

**Figure 1:**
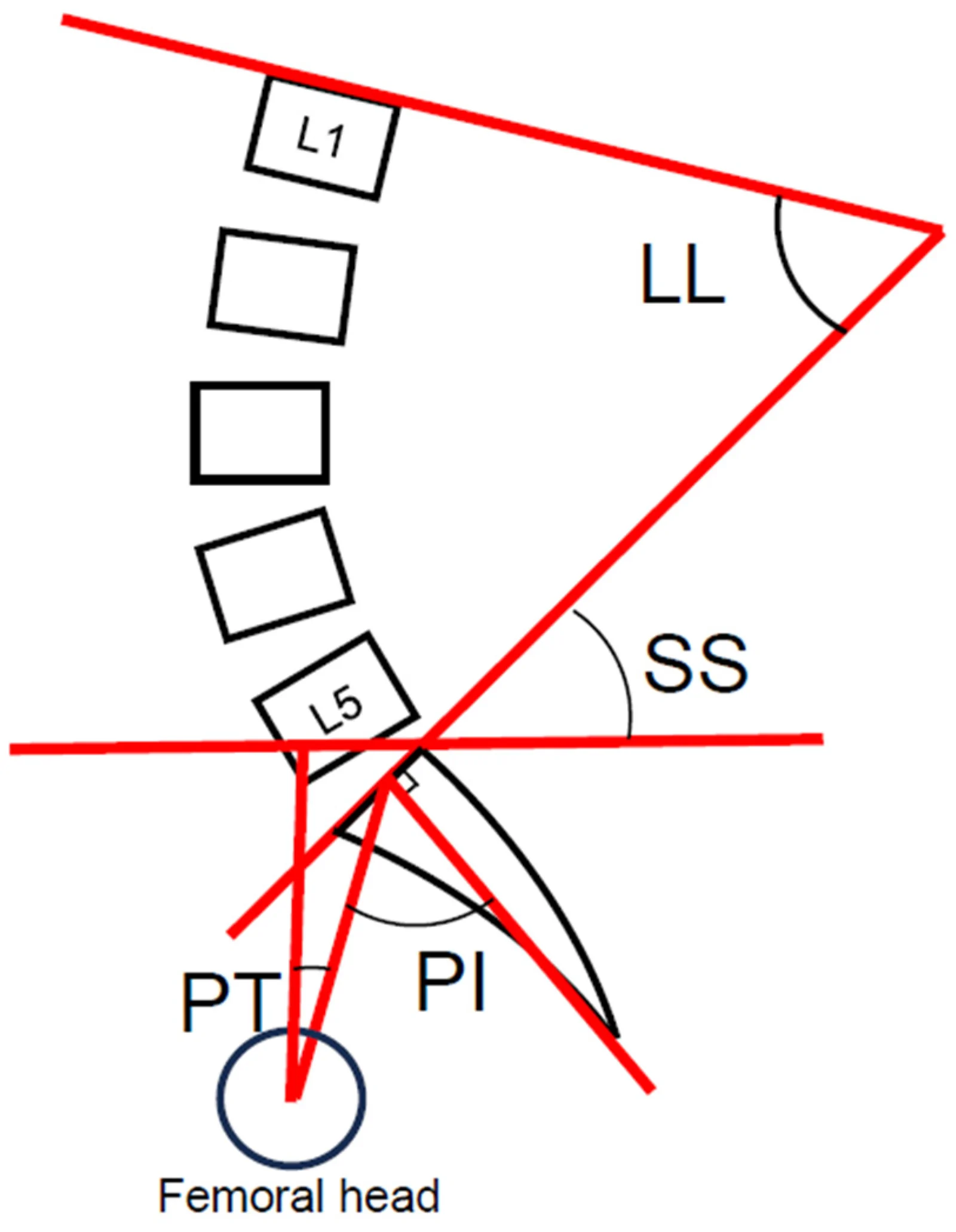
Standing radiographic spinopelvic parameters. LL, lumbar lordosis; SS, sacral slope; PT, pelvic tilt; PI, pelvic incidence.

Standing plain radiographs taken as part of routine assessment were used to measure lumbar lordosis (LL, the angle between the superior endplate of L1 and the superior endplate of the sacrum), pelvic incidence (PI), pelvic tilt (PT) and sacral slope (SS), as illustrated in Figure 1 [11]. PI minus LL (PI–LL) was calculated from the same measurements.

Before the main analysis we assessed the intra-observer reliability of qualitative radiographic grading of structural degeneration [12], such as disc space narrowing and vertebral osteoarthritis, in a random subset of 30 patients. Because of the frequency of multiplanar deformity—structural scoliosis and vertebral rotation—in the older cohort, reproducibility was poor (intraclass correlation coefficient < 0.50). Qualitative grading was therefore abandoned and structural assessment was restricted to the reproducible global alignment parameters listed above.

### 2.5. Primary Outcomes

LBP intensity was rated in four functional situations: (1) at rest, (2) getting up from recumbency, (3) rolling over in bed, and (4) active walking. These four situations were chosen because they are commonly reported by patients with chronic LBP, are readily distinguished by patients themselves, and span both non-weight-bearing and weight-bearing states. We did not measure spinal loading, muscle activity or muscle fatigue, and the four conditions are described here only as the situations in which pain was rated. Pain intensity was quantified using a 100-mm VAS in each condition, where the left anchor represented “No pain” (0 mm) and the right anchor signified “Worst pain imaginable” (100 mm). The total pain score was defined as the cumulative sum of the VAS scores across all four conditions (range: 0–400 mm), and the average pain score was determined by dividing the total score by four. To assess the relative contribution of each functional condition to the overall pain experience, the proportional pain contribution (VAS%) for each phase was calculated as follows:

Rest VAS% = (Rest Pain VAS/Total Pain VAS) × 100

Getup VAS% = (Getting up Pain VAS/Total Pain VAS) × 100

Rolling VAS% = (Rolling Pain VAS/Total Pain VAS) × 100

Gait VAS% = (Gait Pain VAS/Total Pain VAS) × 100

VAS% is a derived index constructed for this study. It has not been validated, and by construction the four values are compositional: they sum to 100%, so an increase in one necessarily reduces the others. VAS% was therefore treated as a descriptive summary of within-patient pain distribution and is reported only alongside the absolute VAS scores, never as a substitute for them.

The primary outcomes were the absolute VAS scores and the VAS% values in each functional phase, compared between the two groups.

### 2.6. Secondary Outcomes

The secondary outcomes involved analyzing correlations between the absolute VAS scores, the VAS% across each functional phase (Rest, Getup, Rolling, Gait), and patient-specific variables—including age and standing radiographic spinopelvic alignment parameters (LL, PT, and the PI–LL value).

### 2.7. Statistical Analysis

To avoid arbitrary sample sizing, a two-stage protocol was used. In a pilot phase, data from 30 consecutive patients (15 per group) were extracted; Gait VAS% was higher in the older group, with an effect size of 0.82. Taking a deliberately conservative estimate of δ = 0.65, an a priori calculation in G*Power (version 3.1.9.7; Heinrich Heine University, Düsseldorf, Germany) with α = 0.05 (two-tailed) and power of 0.90 indicated 51 patients per group, or 102 in total. Consecutive case accumulation then continued until this target was reached. We note that the index used to power the study is the unvalidated derived proportion described above, which limits the precision that should be attributed to this calculation.

Normality was assessed with the Shapiro–Wilk test. Because normality was rejected for most continuous variables, all continuous data are presented as median with interquartile range (IQR) and all between-group comparisons were made with the Mann–Whitney U test. Categorical variables were compared with the chi-square test, or with Fisher’s exact test where expected counts were small.

Statistical significance was defined a priori at *p* < 0.05. Two families of primary between-group comparisons were pre-specified, and a Bonferroni adjustment was applied within each. The first family comprised the four functional pain conditions—rest, getting up, rolling over and walking. These four conditions were compared both as absolute VAS scores and as VAS%; because the two metrics address the same four conditions and VAS% is an algebraic transformation of the same four scores, the family size was taken as the number of conditions (k = 4), giving 0.05/4 = 0.0125. The Total and Average VAS scores are arithmetic summaries of those same four scores and were not counted as additional comparisons. The second family comprised the five standing spinopelvic parameters (LL, PI, SS, PT and PI–LL), giving 0.05/5 = 0.010. Age, sex and BMI were compared as descriptive baseline characteristics and were not part of either primary family. To apply a single uniform criterion across Table 1 and Table 2, the stricter of the two adjusted thresholds (*p* < 0.01) was used throughout. No comparison in either table yielded a *p* value between 0.010 and 0.0125, so the choice between the two thresholds does not affect any reported result.

Univariate associations between pain variables, age and radiographic parameters were assessed with Kendall’s rank correlation coefficient (τ). Multiple linear regression models were then fitted for each functional pain variable. Age, sex, BMI, PT and PI–LL were entered as candidate predictors and reduced by backward elimination, removing the least significant term until all retained terms reached *p* < 0.05; Table 3 reports the final model for each outcome. LL was not entered as a candidate because PI–LL is derived from it and entering both produced severe multicollinearity (variance inflation factor 8.5). Tolerance and variance inflation factors are reported for every retained term. Because backward elimination selects on the same data used for inference, the *p*-values and R^2^ values of the final models are optimistic and should be read as descriptive.

Secondary analyses—correlations and regression models—used the unadjusted threshold of *p* < 0.05 and are explicitly exploratory. Exact *p* values are reported for every comparison so that readers may apply any alternative criterion. All analyses were performed with JASP (version 0.95.4; JASP Team, Amsterdam, The Netherlands).

## 3. Results

### 3.1. Patient Characteristics

A retrospective chart review identified a total of 102 patients with chronic LBP who met all eligibility criteria. The study cohort was divided into two age-based groups: Group M (the middle-aged cohort, <75 years of age; *n* = 51) and Group O (the older cohort, ≥75 years of age; *n* = 51). Detailed patient demographics and standing radiographic spinopelvic parameters are summarized in Table 1. The sex distribution did not differ between the groups (*p* = 0.69), and BMI was lower in Group O, although not at the pre-specified threshold (*p* = 0.027).

LL was significantly lower in Group O than in Group M (median 23.6° vs. 40.4°, *p* = 0.001), and PI–LL was significantly larger (24.3° vs. 13.9°, *p* = 0.005). PT was higher in Group O, but the difference did not reach the pre-specified threshold (27.4° vs. 22.9°, *p* = 0.061). PI (*p* = 0.352) and SS (*p* = 0.063) did not differ significantly between the groups.

### 3.2. Comparison of Pain Conditions Between the Two Groups

Absolute pain intensities (VAS, mm) and relative proportional pain contributions (VAS%) across the four functional phases (rest, getting up, rolling over, and gait) are detailed in Table 2.

Group M reported higher absolute pain than Group O in total (*p* < 0.001) and on average (*p* < 0.001), and in each of the three non-weight-bearing or transitional conditions: rest (*p* < 0.001), getting up (*p* < 0.001) and rolling over (*p* < 0.001). Absolute gait pain did not differ between the groups (median 37.0 vs. 42.0 mm, *p* = 0.730).

The proportional measures followed the pattern implied by these absolute values. Rest VAS% (*p* = 0.004), Getup VAS% (*p* < 0.001) and Rolling VAS% (*p* = 0.004) were higher in Group M, whereas Gait VAS% was higher in Group O (median 59.8% vs. 27.3%, *p* < 0.001). Since absolute gait pain was equivalent in the two groups, the higher Gait VAS% in Group O reflects the lower pain reported in the other three conditions rather than more severe walking pain.

**Figure 2:**
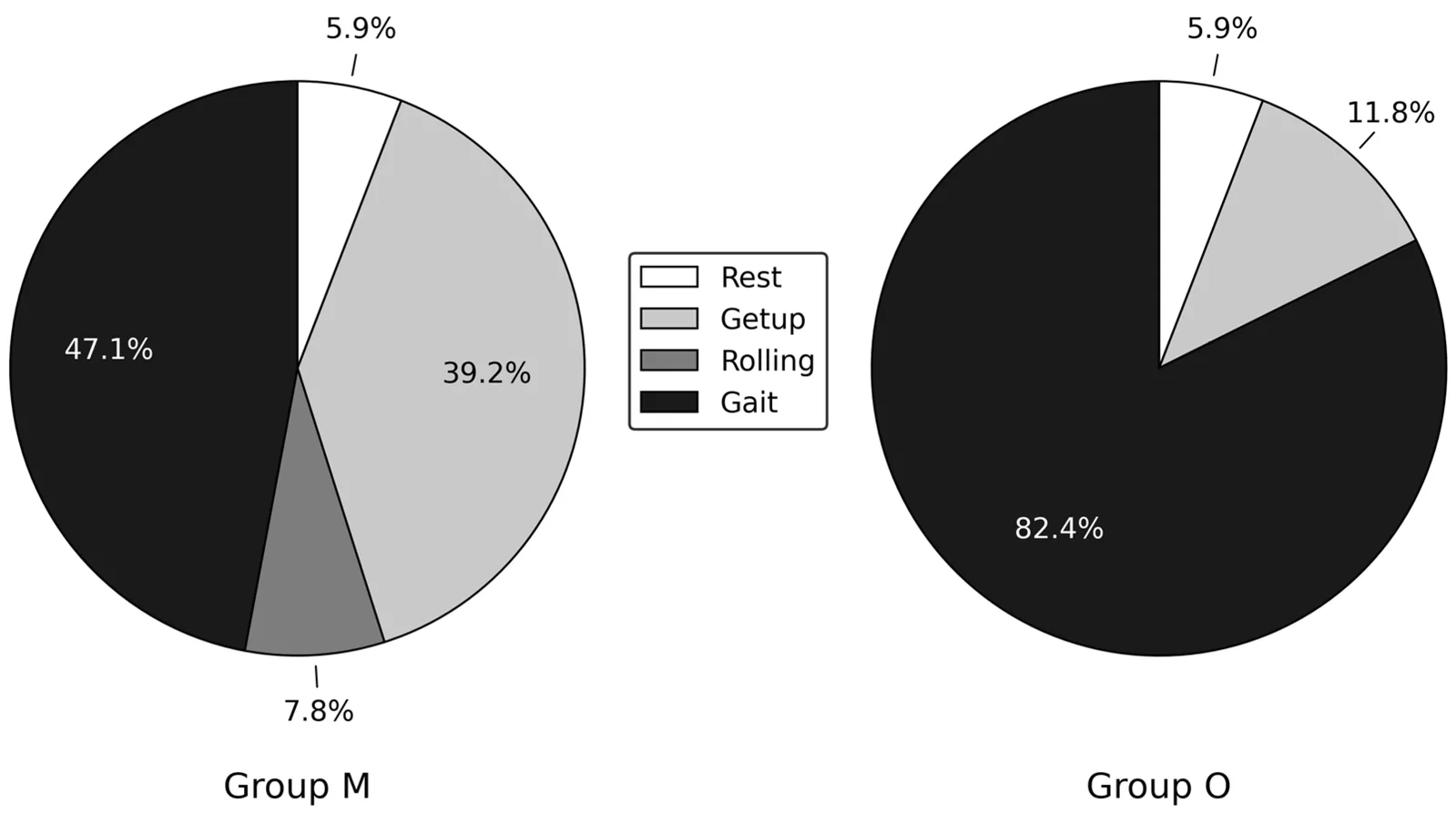
Situation in which patients reported their most severe low back pain, by group (forced single choice). Group M: rest 3 (5.9%), getting up 20 (39.2%), rolling over 4 (7.8%), walking 24 (47.1%). Group O: rest 3 (5.9%), getting up 6 (11.8%), rolling over 0 (0%), walking 42 (82.4%). The distribution differed between the groups (Fisher–Freeman–Halton exact test, *p* < 0.001; walking versus all other situations, Fisher’s exact test, *p* < 0.001).

When patients identified the single situation in which their low back pain was worst, walking was the most common answer in both groups but predominated in the older group: 24 of 51 patients (47.1%) in Group M and 42 of 51 (82.4%) in Group O (Figure 2). The overall distribution differed between the groups (Fisher–Freeman–Halton exact test, *p* < 0.001; walking versus all other situations, Fisher’s exact test, *p* < 0.001). This item is a forced single choice and is presented as a descriptive summary.

### 3.3. Correlations Between Pain Conditions with Age

**Figure 3:**
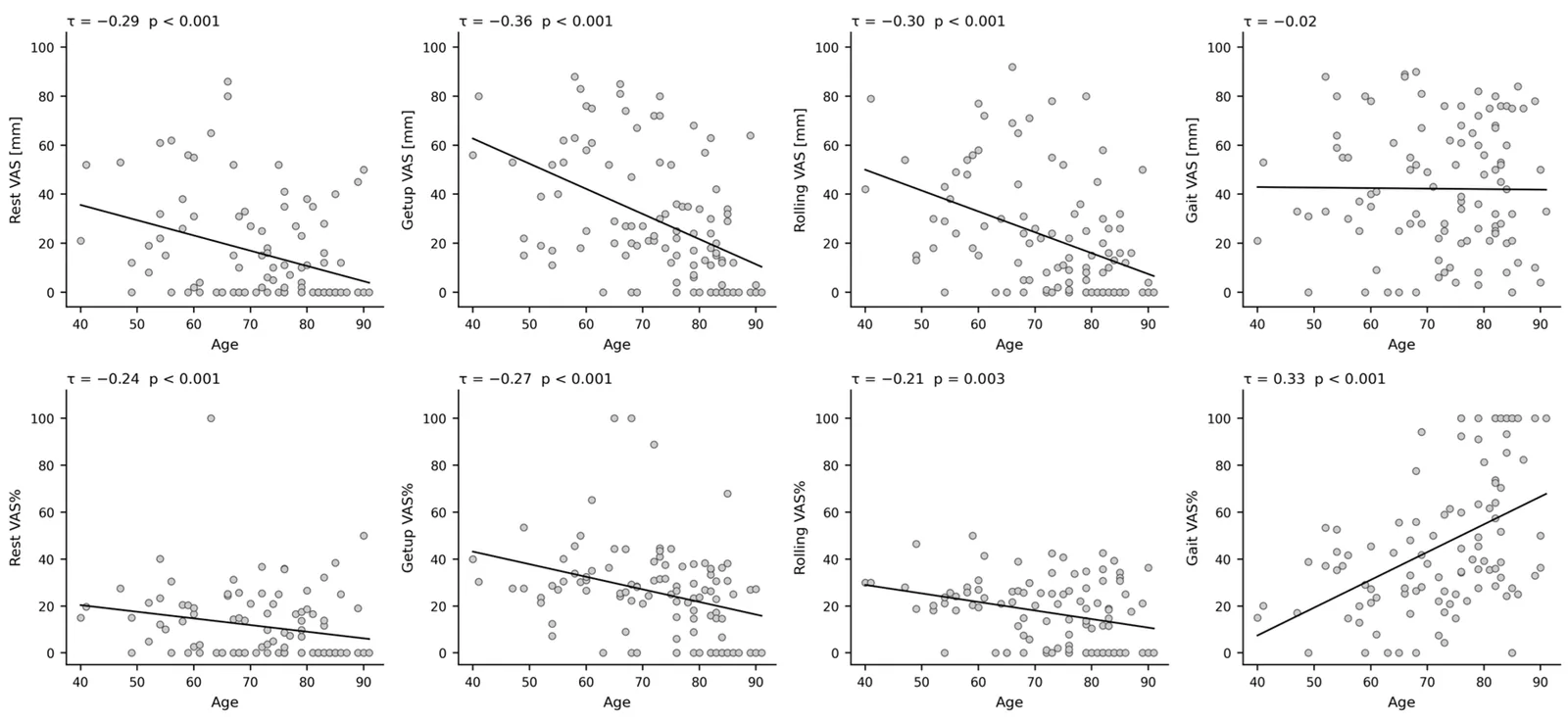
Associations of age with absolute pain intensity (**upper row**) and proportional pain contribution (**lower row**) in each functional phase. Kendall’s τ is shown with exact *p* values where *p* < 0.05; lines are least-squares fits shown for orientation only.

Kendall correlations are shown in Figure 3. Age was weakly to moderately negatively correlated with absolute rest pain (τ = −0.29), getting-up pain (τ = −0.36) and rolling pain (τ = −0.30), and with the corresponding proportional measures: Rest VAS% (τ = −0.24), Getup VAS% (τ = −0.27) and Rolling VAS% (τ = −0.21). Absolute gait pain was not correlated with age (τ = −0.02), whereas Gait VAS% was positively correlated with age (τ = 0.33). These correlations are weak to moderate in magnitude and describe a shift in the relative distribution of pain with advancing age rather than a change in the severity of walking pain itself.

### 3.4. Correlation and Multiple Regression Analyses of Spinopelvic Parameters

**Figure 4:**
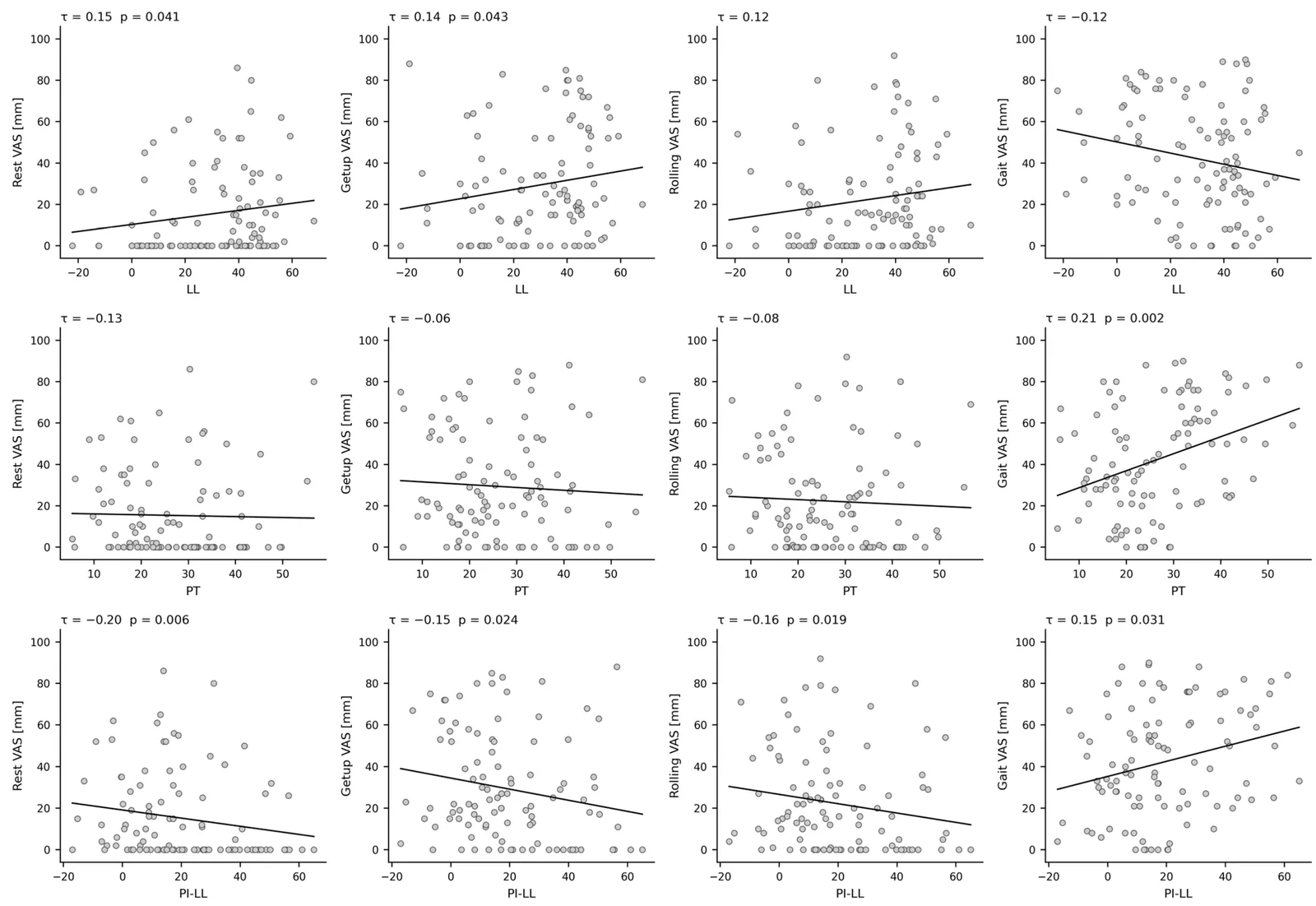
Associations of standing spinopelvic parameters (LL, PT, PI–LL) with absolute pain intensity in each functional phase. Kendall’s τ with exact *p* values where *p* < 0.05.

Univariate associations with absolute pain intensity are shown in Figure 4. Absolute gait pain was positively correlated with PT (τ = 0.21, *p* = 0.002). Absolute rest pain was negatively correlated with PI–LL (τ = −0.20, *p* = 0.006); the remaining associations between absolute pain scores and alignment parameters were weak (|τ| ≤ 0.16) and are shown in Figure 4 for completeness.

**Figure 5:**
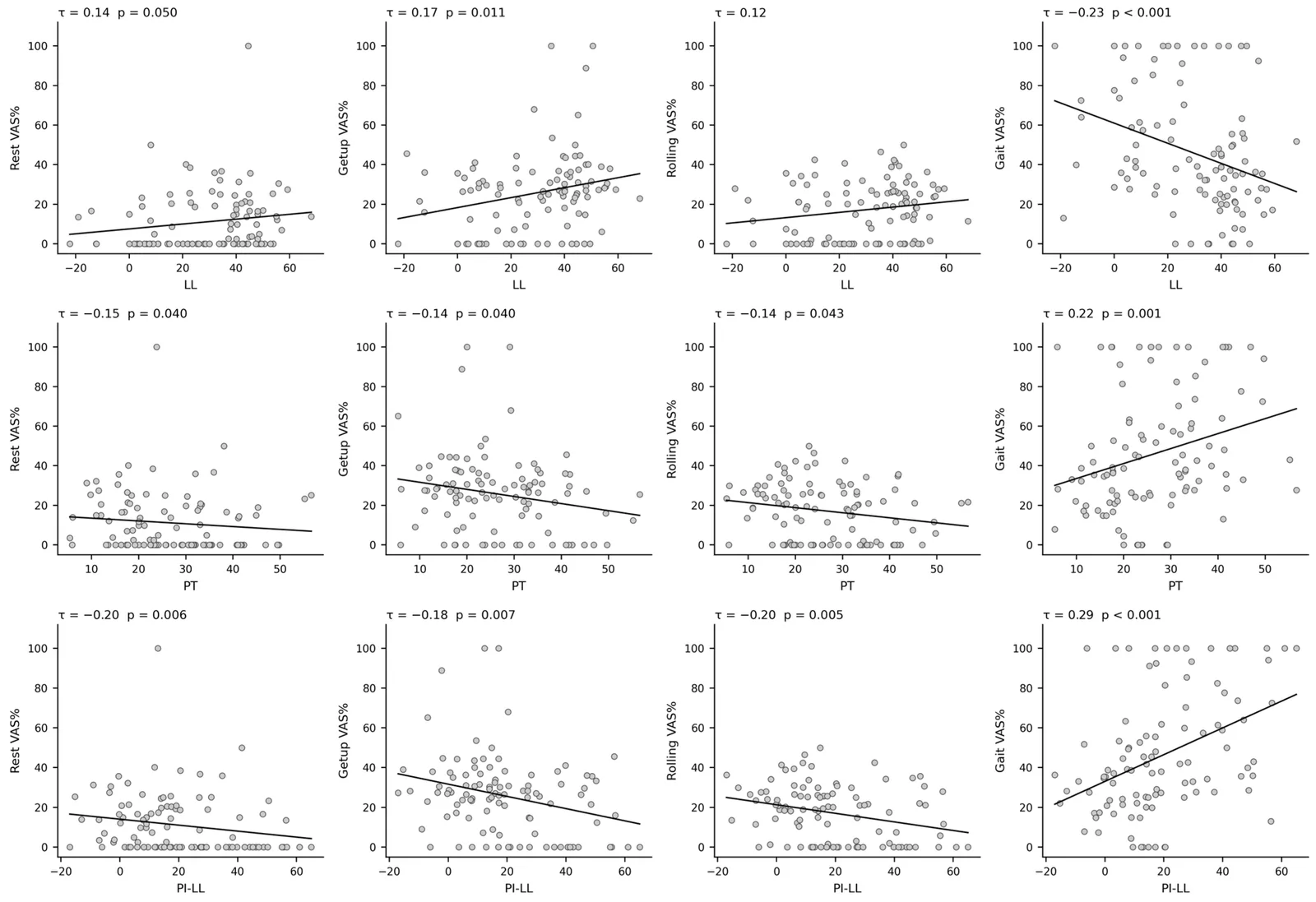
Associations of standing spinopelvic parameters (LL, PT, PI–LL) with proportional pain contribution (VAS%) in each functional phase. Kendall’s τ with exact *p* values where *p* < 0.05.

Associations with the proportional measures are shown in Figure 5. Gait VAS% was positively correlated with PI–LL (τ = 0.29, *p* < 0.001) and PT (τ = 0.22, *p* = 0.001), and negatively correlated with LL (τ = −0.23, *p* < 0.001). Conversely, Rest VAS% (τ = −0.20, *p* = 0.006), Getup VAS% (τ = −0.18, *p* = 0.007) and Rolling VAS% (τ = −0.20, *p* = 0.005) were negatively correlated with PI–LL. All of these coefficients are weak to moderate.

The final regression models are shown in Table 3. For absolute pain intensity, age was the only term retained for rest, getting-up and rolling pain (β = −0.35, −0.47 and −0.42 respectively; all *p* < 0.001), and PT was the only term retained for gait pain (β = 0.35, *p* < 0.001, R^2^ = 0.12). For the proportional measures, age alone was retained for Rest VAS% (β = −0.22, *p* = 0.025), while age and PI–LL were both retained for Getup VAS% (β = −0.25 and −0.21), Rolling VAS% (β = −0.24 and −0.21) and Gait VAS% (β = 0.37, *p* < 0.001 and β = 0.30, *p* = 0.001). Variance inflation factors in all final models were 1.00–1.11.

The model for Gait VAS% had the highest explanatory value of those tested, but its R^2^ of 0.29 indicates that approximately 70% of the variance in proportional walking pain is attributable to factors not captured by these variables.

## 4. Discussion

This cross-sectional study described function-specific profiles of chronic LBP in middle-aged and older patients and examined their association with standing sagittal spinopelvic alignment. Three observations should be kept distinct from one another. First, absolute pain intensity during rest, getting up and rolling over was lower in the older group. Second, absolute pain during walking did not differ between the groups. Third, as an arithmetic consequence of the first two observations, walking accounted for a substantially larger share of the total reported pain burden in the older group. We therefore do not claim that walking pain is more severe in older patients; rather, walking becomes the dominant component of a smaller overall pain burden. This distinction matters, because the four VAS% values are compositional and sum to 100% by definition. We present VAS% only as an exploratory descriptor of the within-patient distribution of pain, and always alongside the absolute VAS scores.

Lower reported pain during rest and during brief postural transitions in older patients is consistent with previous psychophysical work showing that the initial peak sensation of acute pain declines with age whereas the response to prolonged tonic stimulation is relatively preserved [13,14], and with reports that older adults tend to normalise long-standing musculoskeletal symptoms and to under-report baseline discomfort [15,16]. Age-related reductions in peripheral nociceptor density and in the efficiency of endogenous pain modulation have been proposed as contributing mechanisms [17,18]. None of these mechanisms were assessed here: sensory thresholds, conditioned pain modulation, descending inhibition and central sensitisation were not measured, and these explanations are hypotheses generated by our observation rather than tested by it.

The association we observed between a larger standing PI–LL mismatch and a walking-dominant pain profile is compatible with the established principle that sagittal malalignment increases the muscular effort required to maintain upright posture, so that the paraspinal and pelvic-girdle muscles must contract continuously to keep the body within the “cone of economy” [19,20]. A mechanism of this kind would be expected to produce symptoms that emerge during sustained ambulation rather than at rest, which is the pattern we observed. The strength of the association was nonetheless modest: PI–LL correlated with Gait VAS% at τ = 0.29, the standardized β in the final model was 0.30, and that model accounted for 29% of the variance. PI–LL mismatch should therefore be regarded as one contributor among several rather than as a dominant determinant of the functional pain profile. We measured neither dynamic alignment, gait kinematics, paraspinal muscle activity or fatigue, nor walking distance or endurance; our data establish a statistical association between a static standing parameter and a symptom profile, and cannot demonstrate that the proposed mechanical pathway is the operative one.

### Clinical Relevance

One observation follows directly from the design of this study. Walking-aggravated symptoms in older patients are commonly attributed to lumbar spinal stenosis presenting as neurogenic intermittent claudication. Patients with symptomatic stenosis and radiating neurogenic pain were excluded from this cohort in order to isolate axial low back pain. Even so, older patients with a larger PI–LL mismatch reported a temporal pattern that superficially resembles claudication: relatively little pain at rest or during bed mobility, with pain intensifying during walking. This raises the possibility that walking-related exacerbation of axial low back pain in older adults does not invariably indicate a neurogenic cause. We did not assess diagnostic performance, and patients with symptomatic stenosis were excluded by design, so we cannot estimate how often the two patterns coexist in unselected practice and these data support no diagnostic rule. Whether standing sagittal alignment carries diagnostic information in older adults presenting with walking-aggravated low back pain is a hypothesis that would require prospective evaluation in an unselected cohort.

## 5. Limitations

Several limitations of the present study must be acknowledged when interpreting our findings.

First, the design is cross-sectional and retrospective. It supports statements about association only, and cannot establish that the decline of sagittal alignment precedes or produces the observed differences in pain profile.

Second, the principal derived outcome, VAS%, is an exploratory index constructed for this study. It has not been validated, its distributional behaviour is not established, and it is mathematically constrained as described above. The sample size calculation was based on this index, which limits the precision that should be attributed to the power argument. In addition, the regression models were reduced by backward elimination, so their *p*-values and R^2^ values are optimistic.

Third, patients with symptomatic lumbar spinal stenosis were excluded. Because stenosis and neurogenic claudication are common contributors to gait-related symptoms in this age group, the cohort is not representative of the full population of older adults presenting with chronic LBP, and generalisability is correspondingly restricted.

Fourth, several clinically relevant variables were not systematically documented in the source records and could not be included: symptom duration, analgesic use, osteoporosis severity and vertebral compression fractures, hip and knee osteoarthritis, frailty status, physical activity level, and psychosocial measures such as depressive symptoms, pain catastrophising and kinesiophobia. Each of these plausibly influences both pain reporting and walking-related symptoms, and their omission is a substantial source of residual confounding.

Fifth, structural and muscular assessment was limited to standing plain radiographs. We did not grade disc degeneration, facet arthropathy or stenosis severity on MRI, and we did not quantify paraspinal muscle cross-sectional area, fatty infiltration or skeletal muscle mass index. Because paraspinal muscle quality strongly influences the capacity to compensate for sagittal imbalance [21,22], this limits interpretation of the active stabilising subsystem; the lower BMI observed in Group O is a surrogate for overall body composition and cannot substitute for it.

Finally, this was a single-centre study of 102 patients. Prospective, multicentre designs incorporating dynamic motion analysis, quantitative muscle imaging and psychosocial profiling are needed to test the hypotheses that these findings generate.

## 6. Conclusions

In this cross-sectional sample, older patients with chronic low back pain reported lower absolute pain at rest and during postural transitions than middle-aged patients, while absolute pain during walking was similar in the two groups. Walking consequently accounted for a larger proportion of the total reported pain burden in the older group, and this proportion was associated with a larger standing PI–LL mismatch, although the association was modest and the model explained less than a third of the variance. These findings are descriptive and hypothesis-generating. They suggest that a single global or resting pain score may not capture what limits older patients functionally, and that asking separately about pain during walking may add clinically useful information. Prospective studies with dynamic assessment, muscle quantification and psychosocial measures are required before any change to assessment or management strategy can be recommended on this basis.

## Figures and Tables

**Table 1:** Patient Demographics and Radiographic Spinopelvic Parameters.

	M Group (*n* = 51)	O Group (*n* = 51)	*p*-Value
Age (yrs)	65 [12.5]	82 [5.5]	<0.001 **
Sex			0.69
Male (*n*, %)	24, 47%	22, 43%	
Female (*n*, %)	27, 53%	29, 57%	
BMI	23.2 [4.7]	21.5 [3.5]	0.027
LL (°)	40.4 [15.5]	23.6 [32.0]	0.001 *
PI (°)	51.0 [13.9]	47.0 [11.0]	0.352
SS (°)	27.3 [14.9]	21.1 [15.1]	0.063
PT (°)	22.9 [17.1]	27.4 [15.3]	0.061
PI-LL (°)	13.9 [14.4]	24.3 [31.8]	0.005 *

Value: Median and Interquartile Range [IQR]. Normality was rejected for most continuous variables (Shapiro-Wilk *p* < 0.05), so continuous comparisons used the Mann-Whitney U test; sex was compared with the chi-square test. Asterisks denote the pre-specified Bonferroni-adjusted thresholds: * *p* < 0.01, or ** *p* < 0.001. Abbreviation; BMI: Body mass index, LL: Lumbar Lordosis, PI: Pelvic Incidence, SS: Sacral Slope, PT: Pelvic Tilt, PI-LL: Pelvic Incidence minus Lumbar Lordosis.

**Table 2:** Comparison of Absolute Pain Intensity (VAS) and Proportional Pain Contributions (VAS%).

	M Group (*n* = 51)	O Group (*n* = 51)	*p*-Value
Pain VAS (mm)			
Total	132.0 [104.0]	76.0 [53.0]	<0.001 **
Average	33.0 [26.0]	19.0 [13.3]	<0.001 **
Rest	15.0 [32.5]	0.0 [11.5]	<0.001 **
Getup	39.0 [42.5]	12.0 [27.0]	<0.001 **
Rolling	26.0 [42.5]	8.0 [18.0]	<0.001 **
Gait	37.0 [36.5]	42.0 [45.0]	0.730
Pain VAS% (%)			
Rest	13.9 [21.1]	0.0 [13.7]	0.004 *
Getup	30.5 [15.4]	17.2 [27.8]	<0.001 **
Rolling	23.5 [13.7]	11.5 [23.5]	0.004 *
Gait	27.3 [25.3]	59.8 [61.2]	<0.001 **

Each value represents the Median and Interquartile Range [IQR]. All comparisons used the Mann–Whitney U test. Asterisks denote the pre-specified Bonferroni-adjusted thresholds: * *p* < 0.01, or ** *p* < 0.001.

**Table 3:** Multiple Linear Regression Analysis Predictive of Functional Pain Parameters.

Dependent Variables	R^2^	RMSE	AIC	Independent Variables	Standardized β	T-Score	*p*-Value	Collinearity Tolerance	VIF
Rest VAS [mm]	0.13	19.60	900.41	*Age*	−0.35	−3.77	<0.001 **	1.0	1.0
Getup VAS [mm]	0.22	22.88	932.06	*Age*	−0.47	−5.33	<0.001 **	1.0	1.0
Rolling VAS [mm]	0.18	21.84	922.57	*Age*	−0.42	−4.63	<0.001 **	1.0	1.0
Gait VAS [mm]	0.12	24.75	948.02	*PT*	+0.35	+3.75	<0.001 **	1.0	1.0
Rest VAS [%]	0.05	0.15	−96.22	*Age*	−0.22	−2.27	0.025	1.0	1.0
Getup VAS [%]	0.14	0.19	−47.47	*Age*	−0.25	−2.58	0.011	0.90	1.11
				*PI-LL*	−0.21	−2.10	0.038	0.90	1.11
Rolling VAS [%]	0.13	0.13	−119.37	*Age*	−0.24	−2.46	0.016	0.90	1.11
				*PI-LL*	−0.21	−2.13	0.035	0.90	1.11
Gait VAS [%]	0.29	0.26	17.78	*Age*	+0.37	+4.14	<0.001 **	0.90	1.11
				*PI-LL*	+0.30	+3.33	0.001 *	0.90	1.11

Candidate predictors were age, sex, body mass index, PT and PI–LL, reduced by backward elimination (removal at *p* ≥ 0.05); only retained terms are shown. Lumbar lordosis was not entered because PI–LL is derived from it and entering both produced a variance inflation factor of 8.5. Sex and body mass index were not retained in any model. Tolerance and VIF are calculated within each final model. Because the models were selected on the same data used for inference, *p* values and R^2^ are optimistic and should be read as descriptive. Asterisks: * *p* < 0.01, ** *p* < 0.001. Abbreviations: RMSE, Root Mean Square Error; AIC, Akaike Information Criterion; PT, pelvic tilt; PI–LL, pelvic incidence minus lumbar lordosis.

## Data Availability

The data used to this study are available from the corresponding author upon request.

## Abbreviations

The following abbreviations are used in this manuscript:

AIC	Akaike Information Criterion
BMI	Body Mass Index
CLBP/LBP	Chronic Low Back Pain/Low Back Pain
IQR	Interquartile Range
LL	Lumbar Lordosis
MRI	Magnetic Resonance Imaging
PI	Pelvic Incidence
PI–LL	Pelvic Incidence-Lumbar Lordosis (mismatch)
PT	Pelvic Tilt
QOL	Quality of Life
RMSE	Root Mean Square Error
SS	Sacral Slope
STROBE	Strengthening the Reporting of Observational Studies in Epidemiology
VAS	Visual Analog Scale
VAS%	Proportional Pain Contribution of Visual Analog Scale Percentage
VIF	Variance Inflation Factor
